# Reversible Cyclic Voltammetry and Non-Unity Stoichiometry: The Ag/AgBr/Br^–^ Redox Couple

**DOI:** 10.1021/acs.analchem.2c04794

**Published:** 2022-12-22

**Authors:** Haotian Chen, Yuqi Chen, Richard G. Compton

**Affiliations:** Department of Chemistry, Physical and Theoretical Chemistry Laboratory, Oxford University, South Parks Road, OxfordOX1 3QZ, Great Britain

## Abstract

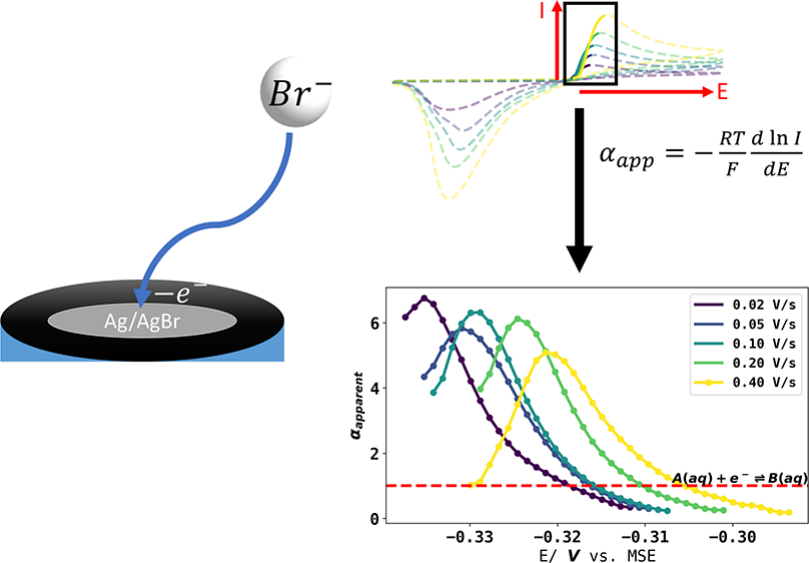

The voltammetry of electrochemically reversible couples in which a soluble reactant is converted into an insoluble product is investigated computationally via simulation and, in the context of the Ag/AgBr/Br^–^redox couple, experimentally. The voltammetric waveshape is characterized and, when analyzed via apparent transfer coefficient analysis, shown to give rise to apparent transfer coefficients very considerably in excess of unity, leading to the generic insight for the characterization of electrode reactions involving solution and solid phase reactants.

## 1 Introduction

Cyclic voltammetry at macroelectrodes is the starting point for almost all fundamental and applied studies in dynamic electrochemistry,^1^ not least since the rigorous quantitative analysis of the resulting voltammograms is well- and long-established via the Randles–Ševčík equations. In particular, for the simple reaction
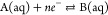
1the Randles–Ševčík equation for a fully reversible process is at temperature *T*:
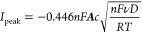
2where *D* and *c* are the diffusion coefficient and bulk concentration of species A, respectively. ν is the scan rate, ***A*** is the geometrical area of the electrode, and *F* and *R* are the Faraday and gas constants, respectively. Similarly, for an electrochemically irreversible reduction process
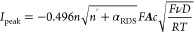
3where α_RDS_ is the transfer coefficient of the rate determining step usually having a value close to 0.5,^1^ and *n*′ is the number of electrons transferred before the rate determining step. In both cases, the scaling of the peak current with the square root of the voltage scan rate fingerprints the diffusional character of the voltammetry. The distinction between reversible and irreversible electrode kinetics is best made via Tafel analysis^2^ of the parts of the voltammetric curve corresponding to ca. 10–30% of the peak current to avoid distortion via mass transport effects,^3^ noting that the IUPAC definition^4,5^ of the reductive transfer coefficient is
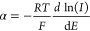
4whereas for Butler–Volmer (BV) kinetics, a plot of ln(*I*) versus *E* is predicted to be linear,^6,7^ although the linearity is only approximate in the case of Hush–Marcus–Chidsey kinetics.^8^ This allows the ready inference of the value of α, whereas for BV kinetics:
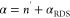
5

In the “reversible” limit for fast electrode kinetics, plots of ln*I* against E, often known as a Tafel plot,^a^, yield an “apparent” transfer coefficient, which for a simple one-electron process takes the value of unity. Note, however, that while simple electron processes give rise to linear or nearly linear Tafel plots, it is recognized that in the case of reversible or quasi-reversible electrode kinetics coupled to homogeneous chemical kinetics, marked deviations occur from the expected value of unity, with the apparent transfer coefficient becoming potential-dependent. Specifically, the presence of a chemical reaction preceding the electron transfer, as in a CE process, gives a reduction in the apparent transfer coefficient and may thus promote an illusion of electrochemical irreversibility, while a following chemical reaction, as in EC and EC_2_ processes, can give rise to apparent transfer coefficients in excess of unity and hence to “super Nernstian” responses^9,10^ of less than the ca. 60 mV per decade expected for a simple one-electron process. These changes are understood to arise from the decrease in the concentrations of species A (CE) or of species B (EC and EC_2_) local to the electrode as a result of the homogeneous chemistry impacting the Nernstian behavior of the redox couple at the electrode–solution interface. Most generally, the value of the transfer coefficient reflects the sensitivity of the current response to the electrical potential and is extensively and most notably used in evaluating candidate redox couples for batteries where the identification of authentic reversibility, rather than the apparent reversibility, is essential.^11^ Burstein highlights the pivotal role of the Tafel plot in establishing the extent of reversibility by suggesting the volt-per-decade-of-current unit might be named after Tafel, i.e., 1 Tafel = 1 V per decade of current, with the symbol Ta. Thus, 1 Ta = 2.3026 V.^12^

In the present paper, and with the aim of identifying the essential conditions for observing particularly large super-Nernstian responses, we consider the voltammetry expected for the case where a simple electrode reaction leads to the formation of an insoluble product and forms a soluble reactant, as for example and in particular in the case of the oxidation of silver in the presence of bromide ion at a silver electrode:
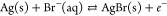
6where the bromide reactant is the only solution phase species, the product being insoluble silver bromide. The redox couple has found use as a (reversible) reference electrode.^13,14^ This then hypothetically represents the extreme case of an EC reaction with ultrafast kinetics in the sense that no soluble products are formed, and so, as will be shown below both theoretically and experimentally, might be expected to lead to extreme Tafel slopes and anomalously high apparent transfer coefficients. Further, we note the link with the highly important and insightful work of Gomez-Gil, Laborda, and Molina^15^ in showing that for non-unity stoichiometry reactions of the general form
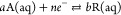
7where *a* ≠ *b*, the reversible Randles–Ševčík eq 1 needs modification to reflect the changed stoichiometry although in all cases the scaling of the peak current with n^3/2^ and ν^1/2^ remains. In particular, Gomez-Gil et al. considered 2:1, 1:2, 3:1, and 1:3 processes as specific examples of (a, b) non-unity stoichiometry focusing exclusively on couples based on solution phase species. In the following, we also establish the appropriate Randles–Ševčík equation as a consequence of exploring the extreme super-Nernstian response of a 0:1 process, where 0 and 1 are the stoichiometric coefficient of the oxidant and reductant. The value of 0 indicates that the redox species is not in the aqueous phase but, for example, in the solid phase, such as AgBr as shown in eq 6.

## 2 Experimental Section

### 2.1 Chemicals and Reagents

Solutions were prepared using deionized water with a resistivity of 18.2 MΩ cm at 298 K (Millipore, Millipak Express 20, Watford, U.K.). All chemicals were of analytical grade and were used as received without any further purification. Potassium bromide (KBr, 99.0%) and potassium nitrate (KNO_3_, 99.0%) were purchased from Sigma-Aldrich, UK.

### 2.2 Electrochemical Apparatus and Methods

Electrochemical measurements were performed using a μAutolab II potentiostat (Metrohm-Autolab BV, Utrecht, Netherlands). A standard three-electrode set-up was used, consisting of a mercury-mercurous sulfate electrode (MSE +0.64 V vs SHE, with saturated K_2_SO_4_, BASi, USA), a graphite rod counter electrode, and a silver macro-disc electrode (diameter of 2.52 mm, homemade) that served as a working electrode. The working electrode were polished using a sequence of progressively smaller (1.0, 0.3, and 0.05 μm) alumina lapping compounds (Bucher, Germany). The electrochemical setup was thermostated at a constant value of 25.0 ± 0.2 °C. High-purity N_2_ flow (BOC Gases PLC, U.K.) was used to remove oxygen from aqueous solutions as needed prior to the electrochemical measurements. Cyclic voltammetric experiments, in which the potential sweep started at −0.55 V vs MSE and swept toward −0.15 V before returning back to −0.55 V, were conducted in a cell containing varying concentrations of potassium bromide (0.2, 0.4, 0.8, 1.2, and 1.6 mM) and 0.1 M potassium nitrate as supporting electrolytes. For each concentration, a study of variable scan rate was performed (20, 50, 100, 200, and 400 mV s^–1^). Prior to each voltammetric measurement, an initial 1 min electrochemical cleaning at −1.45 V vs MSE was conducted.^16^ Each experiment was repeated at least three times.

## 3 Theory

### 3.1 Simulation of Voltammetry

We consider the electrochemically reversible, one-electron oxidation of the surface of a solid Ag macroelectrode in the presence of Br^–^ in an aqueous phase as shown in eq 6.

The mass transport is assumed to be dominated by linear and semi-infinite diffusion, with migration suppressed by the presence of excess supporting electrolyte and convection eliminated by making voltammetric measurements at short timescales. Since only Br^–^ is present in the aqueous phase, the Nernst equation describing the electrochemical reaction is
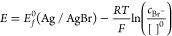
8where [ ]^0^ is the reference concentration of 1 molar, and the activity of both silver and silver bromide considered as pure solids is at unity. *E*_*f*_^0^ is the formal potential of the Ag/AgBr/Br^–^ redox couple. The diffusion equation for the solution phase species Br^–^ is
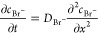
9

For the case of cyclic voltammetry, the spatial boundary conditions at the surface of electrode and the outer boundary of simulation are
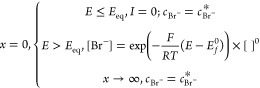
10where *E*_eq_ is the equilibrium potential defined as  and *c*_Br^–^_^*^ is the bulk concentration of bromide.

Linear sweep or cyclic voltammetry increases or decreases the applied potential linearly as a function of time:
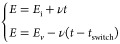
11where *E_i_* is the initial potential and the scan rate, *ν*, is positive for an oxidative sweep. At time *t* = *t*_switch_, the potential reaches *E_v_*, and then the direction of the potential sweep reverses. Equation 11 forms the temporal boundary conditions for eq 9 together with the condition that at *t* = 0, the concentration is *c*_Br^–^_ = *c*_Br^–^_^*^ for all *x*.

Note that the origin of the first boundary condition 10 above lies in the fact that at the start of the experiment, the electrode surface is assumed to be exclusively silver and for potentials below the equilibrium potential, no current flows nor is any silver bromide present as its formation is not thermodynamically possible. Once the potential sweep reaches the equilibrium potential, the reaction switches on and silver bromide is formed. At this point, the equilibrium in eq 8 is established and the surface concentration of bromide is controlled by the second spatial boundary condition; this situation applies as long as silver bromide is present on the electrode surface to maintain the equilibrium. Thus, on the reverse and reductive scans, the model predicts the current until all the AgBr is removed, at which point the current will fall again to zero.

The reaction was simulated using the finite difference method in dimensionless form to increase the generality of the simulations. Table 1 shows the dimensionless parameters used. Note that the definition of the dimensionless potential, θ, is relative to the equilibrium potential, not the formal potential. The dimensionless form of the diffusion equation and the boundary conditions are
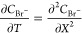
12
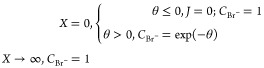
13

**Table 1:** Dimensionless Parameters for Simulation of the Ag/AgBr Electrode^a^

parameter	normalization
concentration	
spatial coordinate	
time	
potential	
scan rate	
current	

a ϵ is the radius of the macroelectrode and *J* is the dimensionless flux defined as . *D*_Br^–^_ and *c*_Br^–^_^*^ are the diffusion coefficients and bulk concentration of bromide, respectively.

Below under Results and Discussion we explore the voltammetry experimentally. To do that requires inference of the surface concentration from experimental voltammetry as described in the next section.

### 3.2 Extraction of Surface Concentrations from Experimental Voltammetric Data

The surface concentration of the electroactive species can be extracted from experimental voltammograms using the backward implicit method.^17^ First, the dimensional voltammogram is converted to its dimensionless form to facilitate the calculations: the dimensional potential is converted to the dimensionless potential, θ′ where θ^′^ = (*E* – *E*_i_)*F*/*RT* and the current, *I*, is converted to dimensionless flux, *J* (Table 1), where *E*_i_ is the initial potential of the voltammetric scan. Detailed implementation and explanation can be found in the “Extracting Surface Concentration” section of the Supporting Information. Figure S1 illustrates and validates the procedure; it shows surface concentrations extracted from one electron reduction of [*Ru*(*NH*_3_)_6_ ]^3+^ as proof of concept since the extracted values coincide with those predicted by the Nernst equation.

## 4 Simulation Methods

Simulations were written in Python, and the diffusion equation was solved using the implicit finite difference method^18^ with an expanding spatial grid to economize on computational resources.^19^ The multi-diagonal sparse matrix was solved using the Newton–Raphson method.^20^ Convergence tests showing the sensitivity of the results to the space step, the time step, and the expanding grid factor are shown in the “Testing and Verification of Simulations” section in the Supporting Information. Convergence data at σ = 100 and 1600 is shown in Figures S2 and S3.

## 5 Simulation Results

**Figure 1:**
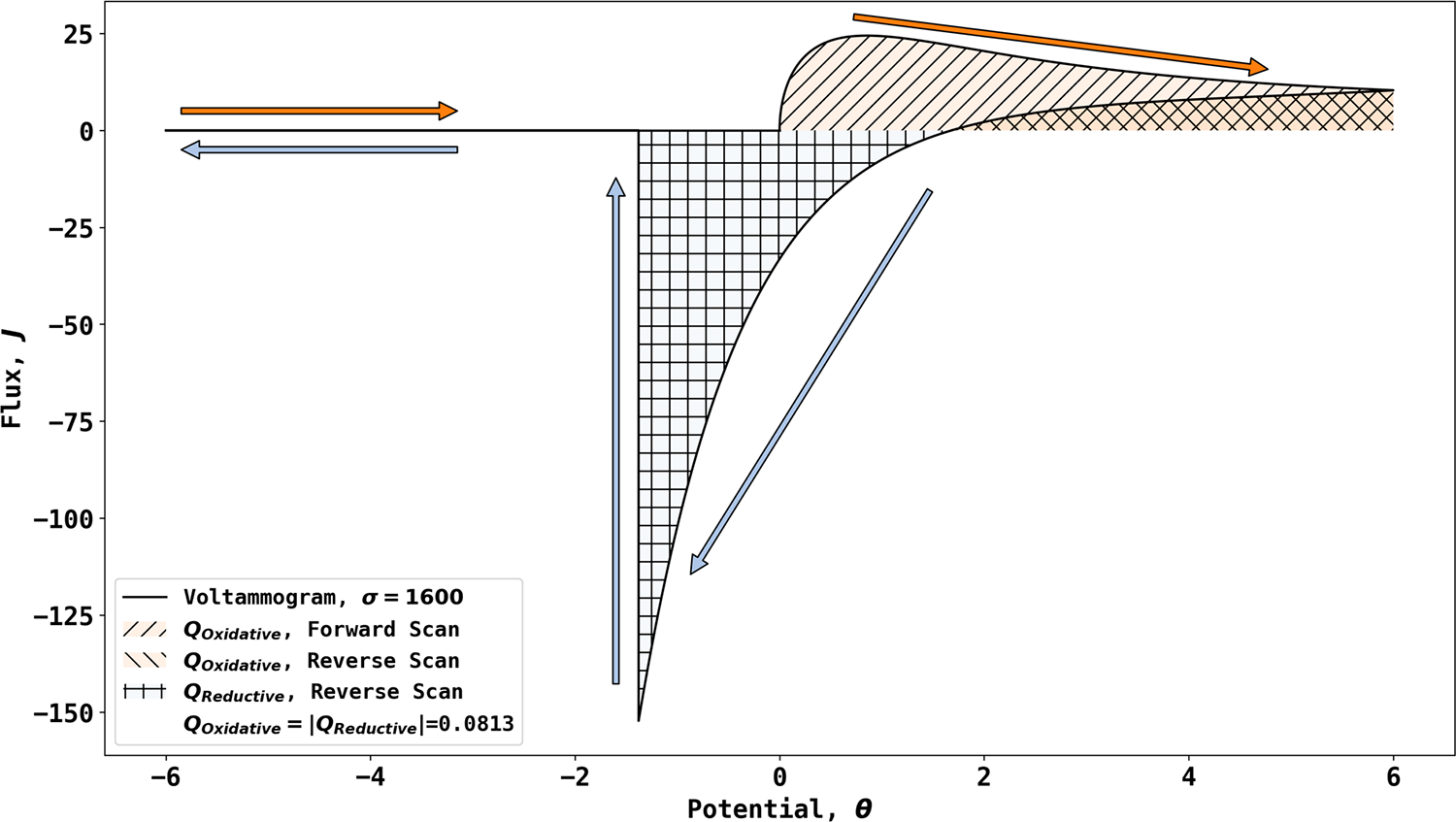
Simulated voltammogram for σ = 1600. The scan starts negative of θ = 0 and reverses at θ = 6, and the reductive current on the reverse scan stops when the reductive charges equals the net oxidative charge on the oxidative scan and that part of the reductive scan between the reversal potential and the potential of zero current at a dimensionless potential of 1.683.

First, voltammetry at a dimensionless scan rate of σ= 1600 was simulated as illustrated in Figure 1. Note that as expected, zero current flows on the initial scan until the potential reaches the equilibrium potential, where *E* = *E*_eq_, so that θ = 0. As the formation of solid silver bromide at the silver electrode then becomes thermodynamically possible, the onset of rapidly increasing currents is observed. The current increases until it passes through a maximum as the available bromide ion in solution is depleted, and the flux drops as the extent of depletion increases. In Figure 1, the oxidative scan is (arbitrarily) reversed at θ = 6. On the reverse scan, oxidative currents continue to flow until the reduction of the deposited AgBr film becomes thermodynamically possible at potentials cathodic of the equilibrium potential, where the latter reflects the concentration of bromide local to the electrode surface, and this value is depleted below the bulk concentration as a result of the oxidative currents passed consuming bromide ions. As the potential of the reverse scan becomes increasingly negative, the current increases more and more until the silver bromide is exhausted, at which point the current returns abruptly to zero and the electrode is again pure silver. Figure 1 illustrates the sum of the oxidative charge in the anodic scan, and that in the reverse scan from the reversal potential to the equilibrium potential matches exactly the total reductive charge passed on the reverse scan at potentials between the equilibrium potential and the potential at which the reductive current drops sharply to zero.

**Figure 2:**
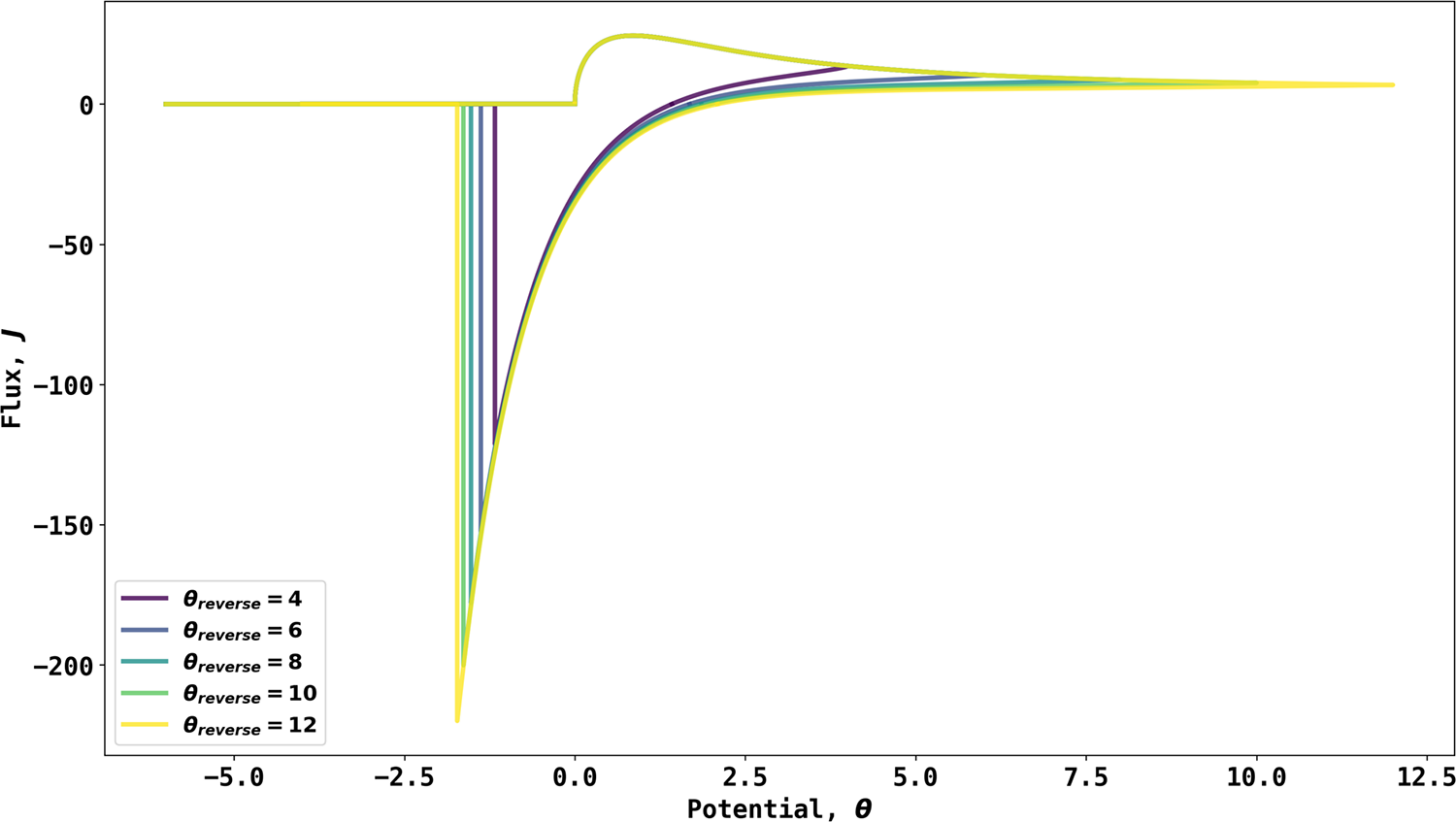
Simulation of voltammetry at σ = 1600 with increasing reversal potentials from 4 to 12.

Figure 2 shows the effect of reversing the potential scan at different potentials in the range 4 to 12 while maintaining the scan rate at σ = 1600. Voltammetry was simulated with increasing reverse potentials from 4 to 12. As is evident in the figure, reversal at increasingly positive potential leads to the greater deposition of AgBr, and hence, the potential at which the current corresponding to the removal of AgBr on the reductive scan becomes increasingly negative. Similarly, the potential on the reverse scan at which reductive currents start to flow becomes increasingly positive, consistent with the greater depletion of bromide ions and the corresponding shift of the local equilibrium potential at the electrode surface.

**Figure 3:**
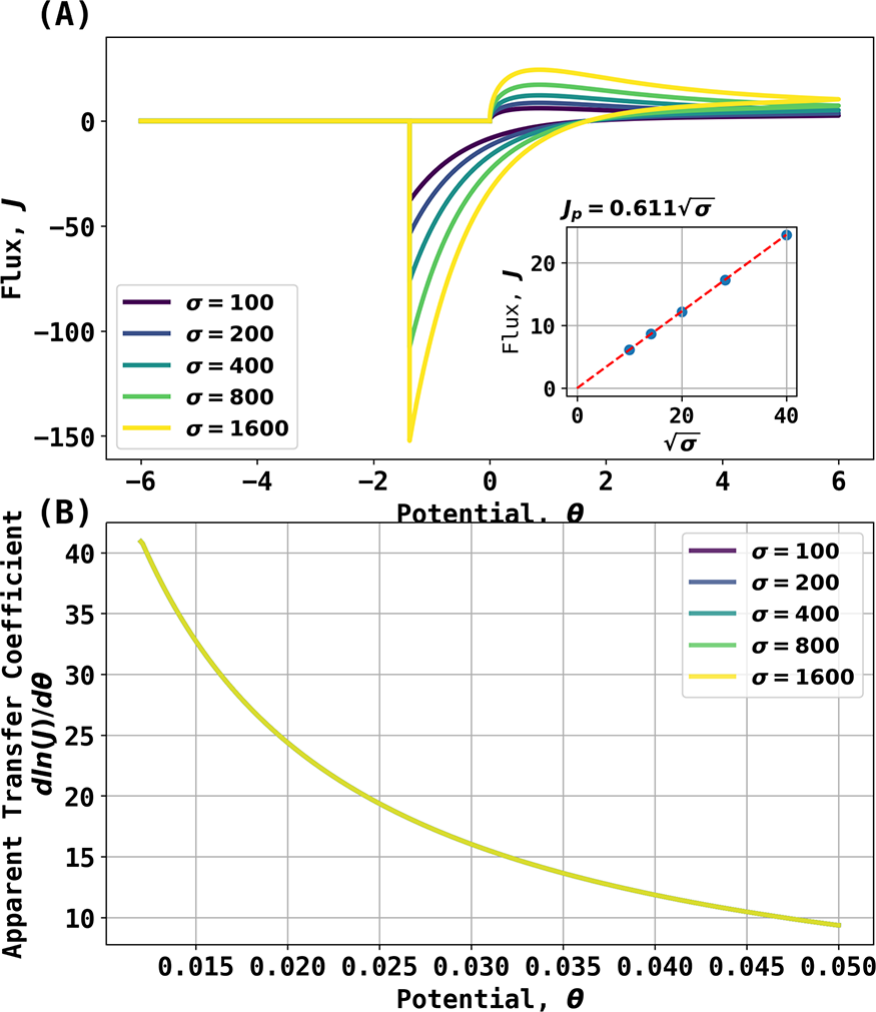
(A) Simulated voltammogram for reaction 6 from σ = 100 to σ = 1600. The black arrow indicates the start and initial direction of the scan. The inlay shows the regression of peak current vs ; (B) apparent transfer coefficient from 20 to 40% of voltammogram before peak current.

Next, simulations were conducted for a range of dimensionless scan rates between 100 and 1600. The results are shown in Figure 3A. The forward (oxidative) voltammetric scan shows a rapidly rising current starting immediately when the voltage scan reaches the equilibrium potential; as before, the current reaches a maximum and then decays as the bromide ion concentration is depleted. After reversal of the potential sweep at a dimensionless potential of 8, a reductive wave is observed as the silver bromide formed on the electrode surface in the forward scan is removed. The sudden drop in current corresponds to the completion of the removal process. Note that the potential at which the current on the reverse scan switches from cathodic to anodic is independent of the scan rate.

As can be seen in Figure 3A, the peak current occurs at a potential of θ_p_ = 0.854 independent of scan rate, while the peak flux increases with the latter. The forward scan peak fluxes, *J*_p_, were regressed with square root of dimensionless scan rates,  as shown in the inlay, which establishes that for the Ag/AgBr/Br^–^ reaction (0,1 stoichiometry),
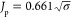
14

In dimensional form, this corresponds to:
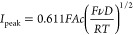
15

This contrasts with the corresponding equation for a one electron process with 1:1 stoichiometry where
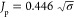
16

Note that the greater sensitivity to scan rate for the 0:1 reaction compared to the 1:1 reaction is related to the more sudden onset of the voltammetric wave in the 0:1 case, which only becomes possible at potentials positive of the equilibrium potential. In contrast, for a 1:1 process, the voltammetric onset is more gradual reflecting the presence of both species, A and B in eq 1, in solution as opposed to the phase change in the system of interest.

The difference between eqs 15 and 16 hints that the electrochemical reaction with 0:1 stoichiometry also exhibits super-Nernstian apparent transfer coefficient slopes. Figure 3B shows the transfer coefficient analysis of voltammograms shown in Figure 3A taken from 20 to 40% of the peak flux by plotting  vs θ*.* Note the extremely large apparent transfer coefficients observed and their potential dependence whereby the apparent transfer coefficient decreases from ∼40 at 20% of the peak flux to ∼10 at 40% of the peak flux, showcasing a super-Nernstian behavior as the apparent transfer coefficient significantly exceeds unity.

## 6 Results and Discussion

**Figure 4:**
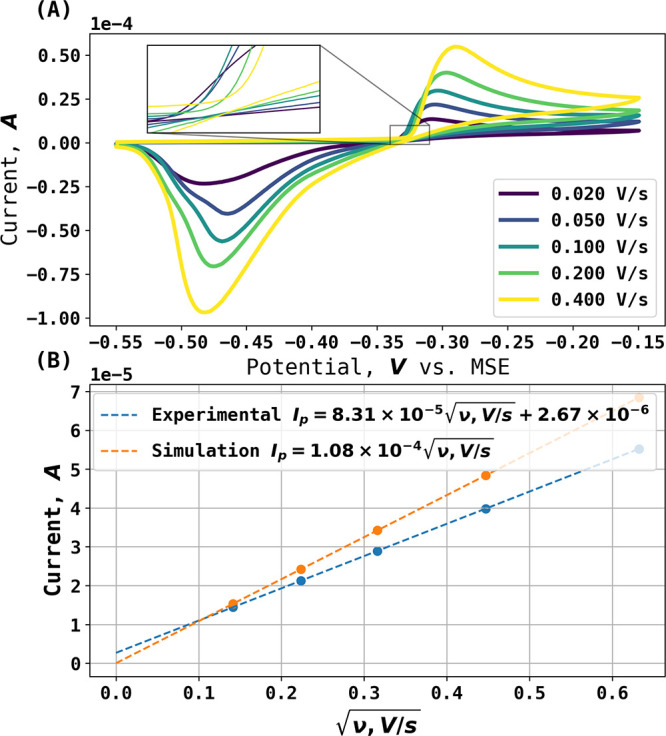
(A) Cyclic voltammograms of 1.6 mM Br- in 0.1 M KNO3 at a Ag macroelectrode of *r* = 1.13 mm as a function of scan rate from 0.02 to 0.4 V/s.The inset shows the oxidative and reductive current near equilibrium potential. (B) Regressing the forward scan peak currents from experiment (blue trace) and simulation (orange trace) against the square root of the scan rate.

Voltammograms were measured using a silver electrode in an aqueous solution of 1.6 mM Br^–^and 0.1 M KNO_3_ at a scan rate from 0.02 to 0.4 V/s starting at a potential of −0.55 V vs the mercury sulfate electrode (MSE) with an oxidative first sweep with reversal of the potential at −0.15 V vs MSE as shown in Figure 4A. The voltammogram shows an oxidative peak corresponding to the formation of AgBr on the forward scan and a reductive peak on the reverse scan related to the reduction of the AgBr to Ag. The equilibrium potential located between the two peaks is estimated as ca. −0.332 ± 0.005 V. Note that the solubility of silver bromide in the medium used is tiny (the solubility product is thought to be ca. 5.0 × 10^–13^ at 298 K) and the experimental conditions are such that the formation of soluble silver complexes of the type AgBr_*n*_^(*n* – 1)^, where *n* is greater than or equal to one, is not expected^21^ as discussed in the “Silver Speciation” section in the Supporting Information. Specifically, the tabulated and visualized speciation data is shown in Table S1 and Figure S4. Variable scan rate experiments were carried out as shown in Figure 4A where an increase in the peak currents with scan rate is evident. The voltammetry in Figure 4A is qualitatively consistent with that reported by Hassan et al,^22^ albeit that the latter was measured under self-supported conditions and is qualitatively that expected for a quasi-reversible process in that both oxidative and reductive peaks are clear, but unlike a fully reversible process, negligible currents flow immediately anodically or cathodically of the equilibrium potential. Comparison of the anodic and cathodic charges passed in the voltammetry suggests that less than 100% of the deposited AgBr is stripped and that the fraction stripped decreases with a decrease in scan rate as shown in the “AgBr Stripping Efficiency” section and Table S2 in the Supporting Information and attributed to mechanical loss of deposited AgBr or, more likely, the formation of AgBr particles, which are electrically isolated from the electrode and therefore not stripped in the reverse scan.

**Figure 5:**
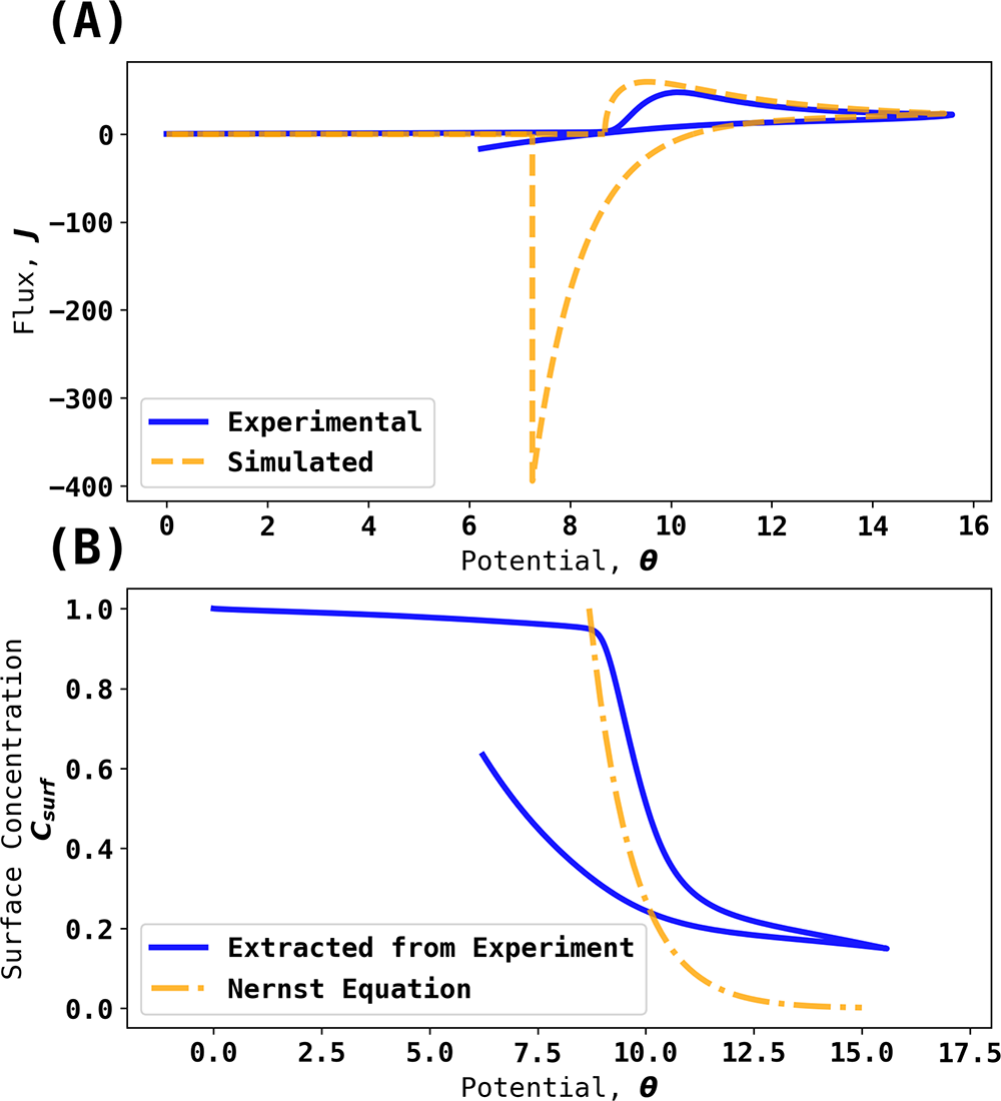
(A) Blue trace: Experimental voltammogram at 0.4 V/s and *c*_Br^–^_^*^ = 1.6 mM. Orange trace: simulated voltammogram. (B) Blue trace: Surface concentration extracted from experiment using backward implicit method. Orange trace: surface concentration predicted via the Nernst Equation with (0:1) stoichiometry.

**Figure 6:**
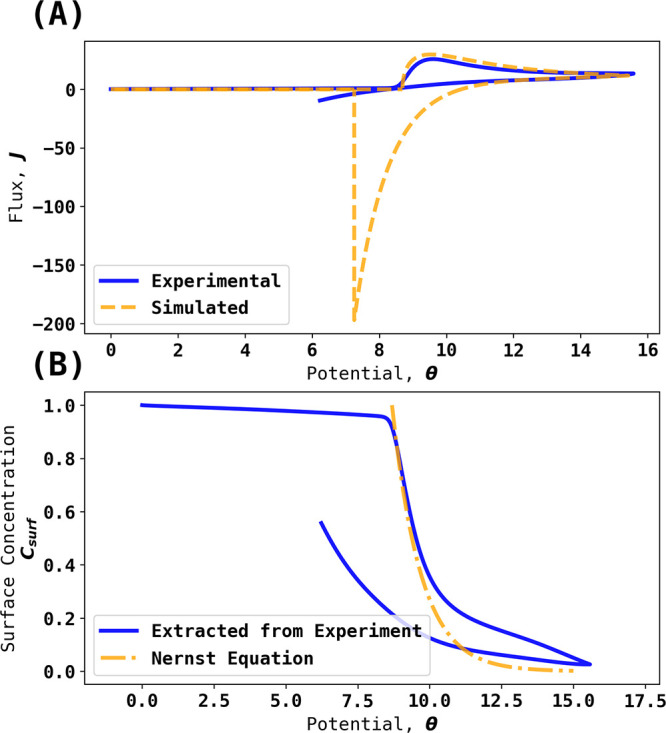
(A) Blue trace: Experimental voltammogram at 0.1 V/s and *c*_Br^–^_^*^ = 1.6 mM. Orange trace: simulated voltammogram. (B) Blue trace: Surface concentration extracted from experiment using backward implicit method. Orange trace: surface concentration predicted via the Nernst Equation with (0:1) stoichiometry.

Figure 4B shows a plot of the peak currents measured in Figure 4A against the square root of the voltage scan rate. The linear, direct dependence is consistent with a diffusional process. Also shown in Figure 4B are the predictions of eqs 2 and 15 above using a value of 2.1 × 10^–9^ m^2^ s^–1^ for the diffusion coefficient of the bromide ion as reported by Kumar et al^23^ measured using microelectrode voltammetry at 298 K. It is evident that the process is diffusional but that the agreement with eqs 2 and 15 is semi-quantitative, with the experimental values seen to be below those predicted theoretically. This can be attributed to the growing silver bromide film on the electrode inhibiting diffusion to the site of electrochemical reaction at triple interface formed by the three phases of silver, silver bromide, and aqueous bromide solution. That said, the quasi-reversible character of the voltammetry was evident. This was further confirmed using the procedure described above for the inference of surface bromide concentrations from the measured currents as a function of potential. Figure 5A shows an experimental voltammogram measured in 1.6 mM Br^–^at a scan rate of 0.4 V/s alongside the simulated voltammogram, which confirms the inferences of quasi-reversibility and restricted diffusion made from the Randles–Ševčík equation plot. Figure 5B shows the corresponding inferred surface concentrations alongside the expectations of the Nernst equation made using an equilibrium potential of −0.323 V. Figure 6A,B shows analogous data but for the slower scan rate of 0.1 V/s. Both Figure 5 and Figure 6 confirm the quasi-reversibility of the voltammetric wave; the fit with the Nernst equation is approximate, and the absolute concentrations observed are lower than expected for full electrochemical reversibility.

Figure 5 highlights the value of the novel simulations with respect to defining the full reversibility or otherwise by comparison with the experiment. The quasi-reversible behavior was clearly established, and this is important if the redox couple is used as a reference electrode. The potential will markedly deviate from ideal behavior if significant currents are drawn through the interface; however, we return to this point further below.

Returning to Figure 5, comparison of the simulated reversible behavior with the experimental cathodic stripping peak showed that considerable overpotential is required to reduce the deposited silver bromide. Data in the “AgBr Thickness” section in the Supporting Information shows that the average thickness of the deposited AgBr film is of the nanometer scale. Table S3 shows the parameters used to calculate the thickness, Figure S5 shows the AgBr average thickness progressing with voltammetric scans when *c*_Br^–^_^*^ = 1.6 mM and *ν* = 400 mV/s. Table S4 tabulates the estimated average AgBr deposit thickness for scans at different concentrations and scan rates. In reality, a non-uniform porous film is likely. Further modeling based, for example, on Butler–Volmer kinetics would need to recognize that the site of nucleation and growth or stripping of the deposit would likely be located at the triple interface formed between solid Ag, solid but poorly conductive AgBr, and the bromide in aqueous phase as well as that of uncertain dimensions. Thus, the simulation of the kinetically controlled quasi-reversible voltammogram is challenging since the use of average AgBr coverages with uniform reactivity such as used for sub-monolayer molecular layers^24^ is likely to be unrealistic when a new phase, such as that of AgBr, is formed.

**Figure 7:**
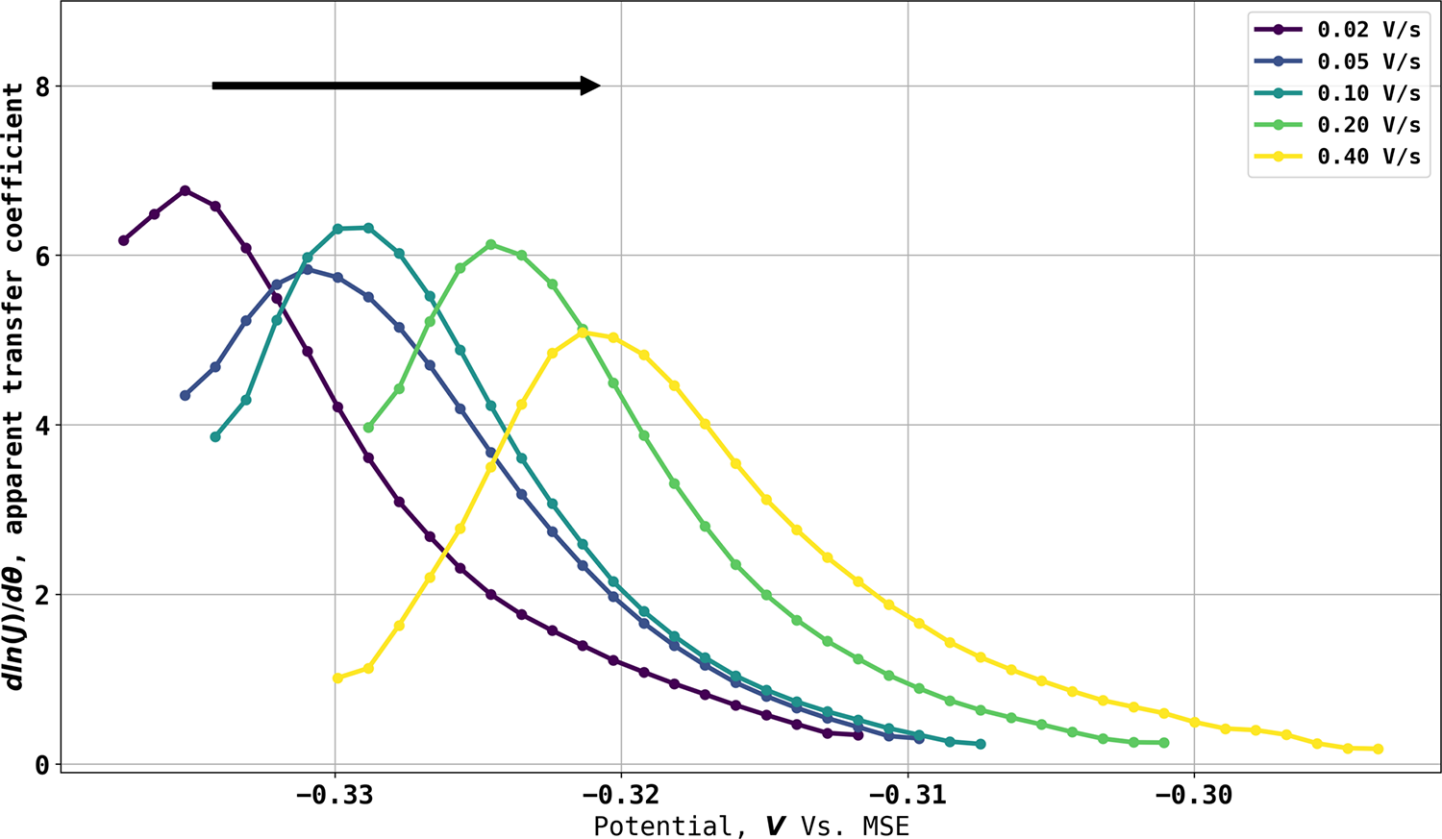
Tafel slopes for voltammograms shown in Figure 4A at scan rates from 0.02 to 0.4 V/s. The Tafel slopes are extracted from part of the voltammogram before the oxidative peak: the starting potential corresponds to 10% of peak current, and the ending potential corresponds to 90% of peak current. The black arrow indicates the direction of the scan.

Given that the partial electrochemical reversibility seen experimentally would likely allow the possibility of super Nernstian responses, attention was next given to transfer coefficient (Tafel-like) analysis of the experimental voltammetry as shown in Figure 4A and the results of the transfer coefficient analysis are shown in Figure 7. The transfer coefficient analysis was performed by analyzing a fraction of the voltammogram before the forward scan peak current and in this case, from 10% to 90% of peak current. The dimensional voltammograms were then converted to dimensionless form, and the apparent transfer coefficients are estimated as . It is evident that the latter are both potential-dependent and take values very considerably in excess of the value of unity expected for a simple one electron 1:1 process, validating the hypothesis formulated in the Introduction that extreme behavior in terms of apparent transfer coefficients is expected for 0:1 process with reversible, or in the case studied, even quasi-reversible voltammetry shows super-Nernstian responses. This is an important new insight. The silver–silver bromide couple has been widely and extensively used as a successful reference electrode for many years.^14^ As noted above, the couple is voltammetrically quasi-reversible, which at first sight is a disadvantage. However, the intrinsic high sensitivity of the current response to the potential shown above to be characteristic of the 0:1 process more than offsets the effects of the quasi-reversibility so that experimentally apparent transfer coefficients in excess of unity are seen. Since a key property of a good reference electrode is holding a near-fixed potential even when passing a current, it can be seen that this confers significant advantage on reference electrodes based on 0:1 processes where a solution phase species is in equilibrium with a solid phase, as for example in silver–silver chloride, calomel, and Ag–Ag^+^ electrodes in addition to the silver–silver bromide electrode as compared to, say, the reference electrodes based exclusively on solution-phase species.

## 7 Conclusions

The Randles–Ševčík equation for a 0:1 stoichiometry has been established as  in dimensionless form. Simulation of the voltammetry showed that the apparent transfer coefficients significantly exceed unity, displaying values as high as 40. The simulated expected super-Nernstian behavior is observed in the linear sweep voltammetry of the oxidation of bromide ions at a silver electrode despite the system displaying quasi-reversible behavior with apparent transfer coefficients as high as 7. The paper thus confirmed, both computationally and experimentally, super-Nernstian behavior for redox systems of 0:1 stoichiometry and concluded, generically, that for electrode processes where soluble solution redox species form solids, ultrahigh transfer coefficients far exceeding unity can be observed. The latter observation has implications for the design of reference electrodes best able to provide a near-constant electrode potential despite the passage of significant current. Electrodes based on redox couples of 0:1 stoichiometry are intrinsically more robust in this respect than couples based entirely on solution phase species.

## Supporting Information Available

Extracting surface concentrations; testing and verification of simulations; silver speciation; AgBr stripping efficiency; AgBr deposit thickness (PDF)

## Footnotes

a The original Tafel plot follows the equation: ϵ = *a* + *b* log *J*, where ϵ is the potential and *J* the current density.^2^
