# Supplementary material for: Reversible Cyclic Voltammetry and Non-Unity Stoichiometry: The Ag/AgBr/Br– Redox Couple
Source: Anal Chem. 2022 Dec 22;95(2):1663–70. doi: 10.1021/acs.analchem.2c04794 (PMC9850347; doi:10.1021/acs.analchem.2c04794)
Supplement: Supplementary file 1 — ac2c04794_si_001.pdf [file ac2c04794_si_001.pdf]

Supporting Information - Reversible Cyclic Voltammetry and Non-unity Stoichiometry: The Ag/AgBr/Br^- Redox Couple

Haotian Chen[1], Yuqi Chen[1], Richard G. Compton*

[1] Joint first authors

Department of Chemistry, Physical and Theoretical Chemistry Laboratory, Oxford University, South Parks Road, Oxford OX1 3QZ, Great Britain

* Corresponding author

Email address: Richard.compton@chem.ox.ac.uk (R. G. Compton)

1 Extracting surface concentrations

This section explains the methodology to extract surface concentrations from experimental voltammograms using the method of Henstridge.[1]

First, the voltammogram is converted to dimensionless form using the table shown in the text except for the definition of the dimensionless potential which is noted in the main text. The dimensionless voltammogram then takes the form of dimensionless flux, J, versus dimensionless potential.

In modelling the voltammetry, the flux at time step k, J^k,is approximated by $J^k = \left(\frac{C^k_{X=0} - C^k_{X=\Delta X}}{\Delta X}\right)$, so rearrangement gives $C^k_{X=\Delta X} - C^k_{X=0} = J^k \Delta X$. Note that $C^k_{X=0}$ is the surface concentration of the electroactive species, $C^k_{X=\Delta X}$ is the concentration near electrode surface at $X = \Delta X$, where ΔX is a small spatial step. Using the Newton-Raphson method, $C^k_{X=0}$ can be estimated from a knowledge of J^k.

The backward implicit method solves the diffusion equation in finite difference form:

$$\frac{C^k_i - C^{k-1}_i}{\Delta T} = \frac{C^k_{i-1} - 2C^k_i + C^k_{i+1}}{(\Delta X)^2} \# (1)$$

where i and k represents the current spatial and time steps. The equation can be rearranged as:

$$C^{k-1}_i = -\lambda C^k_{i-1} + (1 + 2\lambda) C^k_i - \lambda C^k_{i+1} \# (2)$$

where $\lambda = \frac{\Delta T}{(\Delta X)^2}$.

From the boundary condition it can further be inferred that $C^k_{X=\Delta X} - C^k_{X=0} = J^k \Delta X$

Using the Backward Implicit method, a tridiagonal sparse matrix is constructed to solve for the concentration profile at time step k:

$$\begin{bmatrix} 1 & 1 & 0 & 0 & 0 & 0 & 0 & 0 \\ \alpha & \beta & \gamma & 0 & 0 & 0 & 0 & 0 \\ 0 & \alpha & \beta & \gamma & 0 & 0 & 0 & 0 \\ 0 & 0 & \alpha & \beta & \gamma & 0 & 0 & 0 \\ 0 & 0 & 0 & \dots & \dots & \dots & 0 & 0 \\ 0 & 0 & 0 & 0 & \dots & \dots & \dots & 0 \\ 0 & 0 & 0 & 0 & 0 & \alpha & \beta & \gamma \\ 0 & 0 & 0 & 0 & 0 & 0 & 0 & 1 \end{bmatrix} \times \begin{bmatrix} C_0^k \\ C_1^k \\ C_2^k \\ C_3^k \\ \dots \\ \dots \\ C_{n-2}^k \\ C_{n-1} \end{bmatrix} = \begin{bmatrix} J^k \Delta X \\ C_1^{k-1} \\ C_2^{k-1} \\ C_3^{k-1} \\ \dots \\ \dots \\ C_{n-2}^{k-1} \\ 1 \end{bmatrix} \#(3)$$ where $\alpha = -\lambda, \beta = 1 + 2\lambda, \gamma = -\lambda$.

By solving the concentration profile sequentially for increasing k, the surface concentration as a function of time or potential can be obtained.

To validate our implementation of the inversible method prior to application to the (0,1) system of interest, the experimental voltammogram for the one electron reduction of $[Ru(NH_3)_6]^{3+}$ to $[Ru(NH_3)_6]^{2+}$ was analysed and the resulting surface concentrations compared with Nernst equation. The system is well characterised [1]. The experiment was performed at a scan rate of $0.5\ V/s$ with $1\ mM\ [Ru(NH_3)_6]^{3+}$ in $0.1\ M\ KCl$ electrolyte and with a glassy carbon electrode ($r = 1.5\ mm$) as working electrode. From literature, the diffusion coefficient of $[Ru(NH_3)_6]^{3+}$ in such conditions is $8.43 \times 10^{-10} m^2 s^{-1}$.[2] The extracted surface concentrations shown in Figure S 1B agreed quantitatively with the Nernst equation when θ>10, while the small false apparent negative concentration extracted when $\theta < 10$ result from small background currents flowing in the experimental voltammetry. We conclude that the backward implicit model can quantitatively extract the surface concentrations of electroactive species from experimental data.

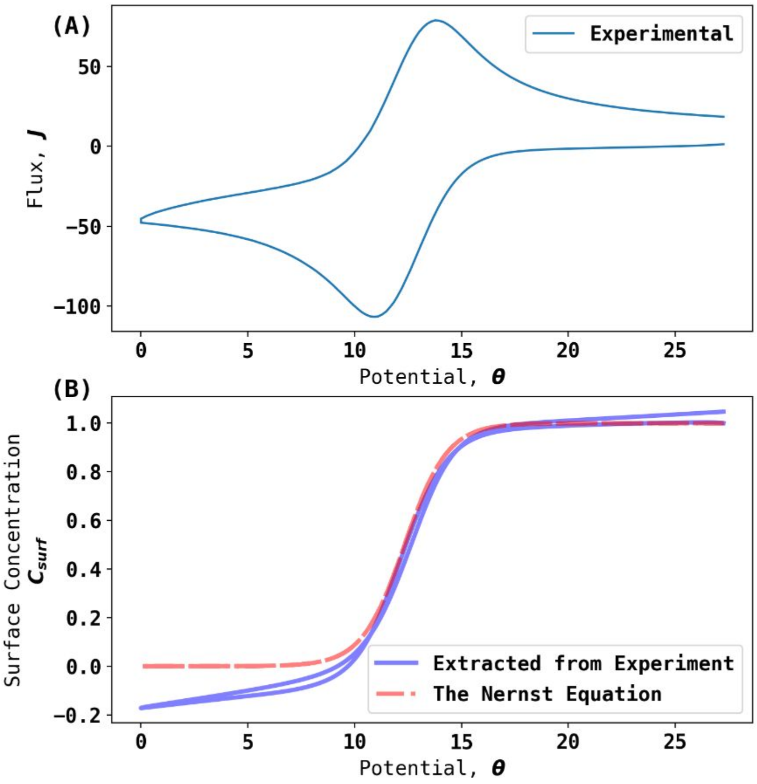

Figure S 1. (A) Dimensionless form of a voltammogram of reduction of $1\ mM\ [Ru(NH_3)_6]^{3+}$ in $0.1\ M\ KCl$ on a glassy carbon electrode. (B) The extracted surface concentration is compared with the Nernst equation with 1:1 stoichiometry.

2 Testing and verification of simulations

Simulation of the electrochemical reaction model utilized expanding space grids[3] to reduce computational time. If the scans start and finish at the same potential, the time for the full scan is $T_{sim} = \frac{2\,|\theta_i - \theta_{switch}|}{\sigma}$, where θ_i and θ_{switch} are the dimensionless starting and switching potentials respectively. σ is the dimensionless scan rate. The maximum spatial distance of the simulation in solution is taken as $X_{sim} = 6\sqrt{T_{sim}}$ [4]. The accuracy of the simulations, were dependent on the spatial step size and time step size. The spatial step size in an expanding grid depends on the initial step size, h_0, and the expanding grid factor, ω, according to the following definition:

$h_i = X_{i+1} - X_i = h\omega^i$where X_i is the dimensionless spatial coordinate at point i. In simulation, h_0 was related to X_{sim} by $h_0 = \lambda X_{sim}$ where λ is a simulation factor determining the initial spatial step. To test convergence of the simulation, λ was varied from 10^{-9} to 10^{-5} and ω varied from 1.01 to 1.2. Since a uniform time grid was used, $d\theta$ was varied from 10^{-5} to 10^{-2} while σ was fixed at 1600 and 100, the highest and lowest scan rates presented in the paper.

The voltammograms generated were validated by comparing the forward scan peak flux, J_p and peak potential, θ_p at different values of λ, $d\theta$ and ω as shown in Figure S 2 and Figure S 3. The simulation parameters at $\lambda = 10^{-7}$, $d\theta = 10^{-4}$ and $\omega = 1.05$ results in converged peak flux at $J_p \approx 24.42$ and peak potential at $\theta_p = 0.85$ when $\sigma = 1600$. At a $\sigma = 100$, using the same set of parameters, $J_p \approx 6.15$ and $\theta_p = 0.85$. The converged peak fluxes and peak potentials using the simulation parameters mentioned above validated the simulations.

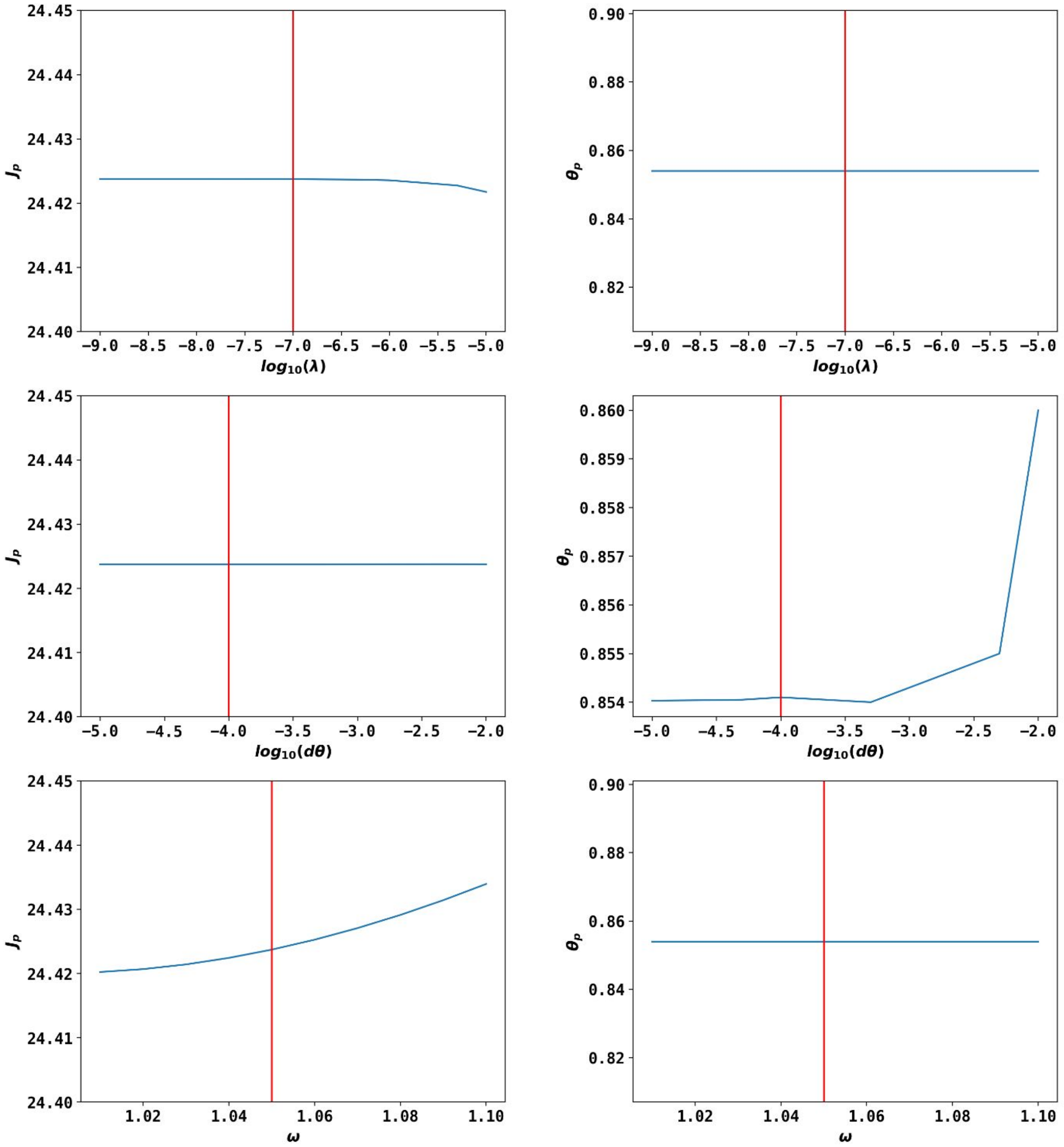

Figure S 2. Convergence test as discussed in the text of the SI when $\sigma = 1600$*, the highest scan rate analysed in text. The left column of three figures shows the responses of the peak flux* J_p *at different values of* λ, $d\theta$ *and* ω*; The right column shows the corresponding responses of the peak potential,* θ_p*. The red line shows the values at the three parameters selected for use in the simulations reported in the text.*

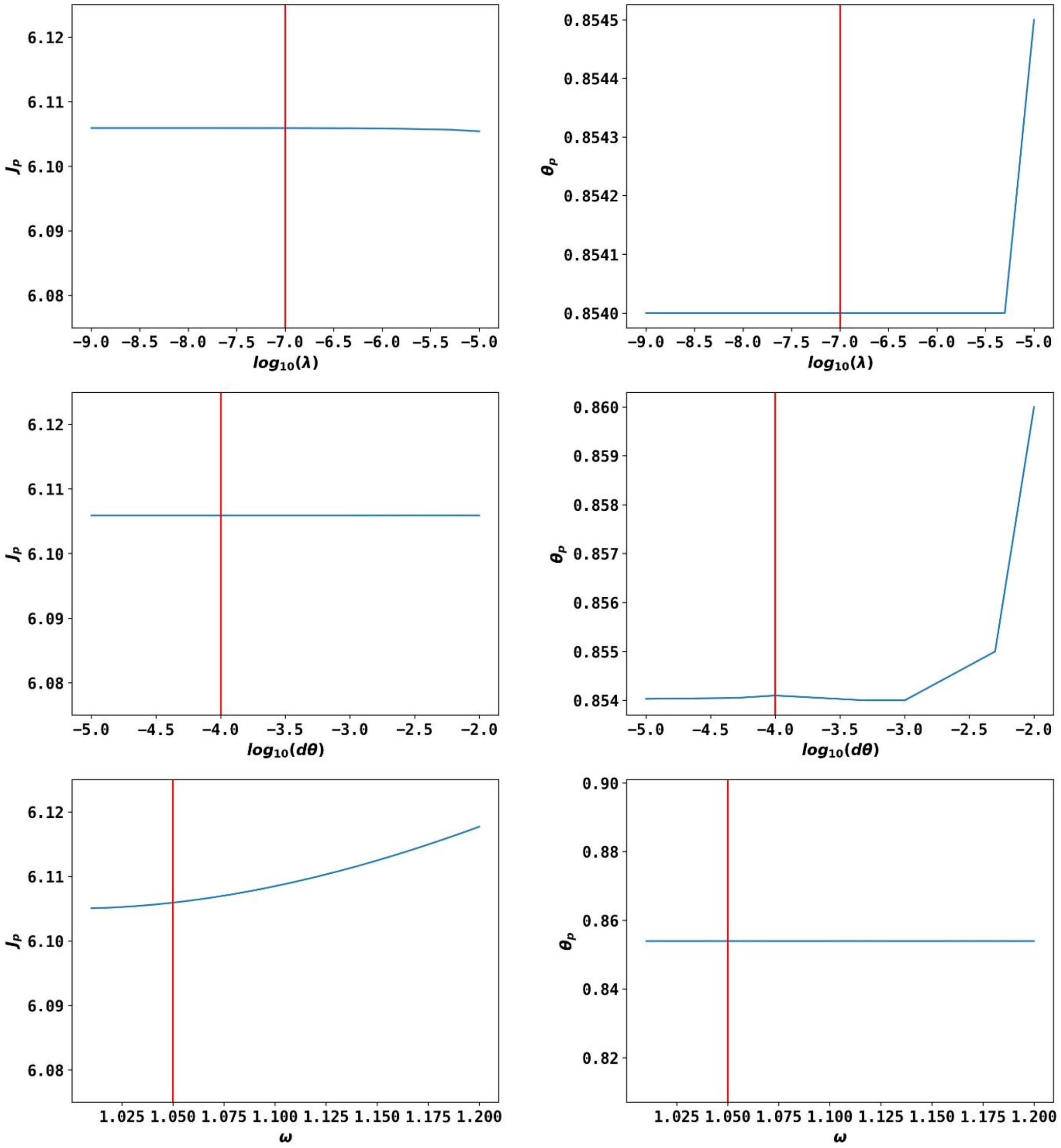

Figure S 3. Convergence test as discussed in the text of the SI when $\sigma = 100$, the lowest scan rate analysed in text. The left column of three figures shows the responses of the peak flux J_p at different values of λ, $d\theta$ and ω; The right column shows the corresponding responses of the peak potential, θ_p. The red line shows the values at the three parameters selected for use in the simulations reported in the text.

3 Silver speciation

To clarify the solution conditions relating to the voltammetry reported in the main text, the speciation of silver (I) in the solutions of interest were calculated using Hydra/Medusa.[5] The chemical equilibria considered were as follows:

$$AgBr(s) \rightleftarrows Ag^+(aq) + Br^-(aq) \qquad 4.1$$
$$AgBr(s) \rightleftarrows AgBr(aq) \qquad 4.2$$
$$AgBr(s) + Br^-(aq) \rightleftarrows AgBr_2^-(aq) \qquad 4.3$$
$$AgBr(s) + 2Br^-(aq) \rightleftarrows AgBr_3^{2-} \qquad 4.4$$

Figure S 4 shows the resulting distribution diagram of the different soluble silver species, $AgBr_n^{(n-1)-}$, as a function of the total bromide concentration. Note that the x-axis scale is logarithmic. The corresponding percentages of different species (Ag^+, AgBr, and $AgBr_2^-$) at different concentrations are summarized by Table S 1. For voltammetry in a 1.6 mM bromide solution, AgBr(aq) accounts for 76% of the silver (I), while $AgBr_2^-$ and Ag^+ are 11% and 13% respectively. Note that $AgBr_3^{2-}$ is not present in solutions with low concentration of bromide[6].

Table S 1. Concentration of Ag^+, $AgBr$ and $AgBr_2^-$ ion as a function of total concentration of Br^-.

$c^*_{Br^-}$, mM	$Ag^+(aq)$, %	$AgBr(aq)$, %	$AgBr_2^-(aq)$, %
0.2	11%	76%	13%
0.4	32%	64%	3.2%
0.8	28%	67%	4.1%
1.2	20%	74%	7.0%
1.6	13%	76%	11%

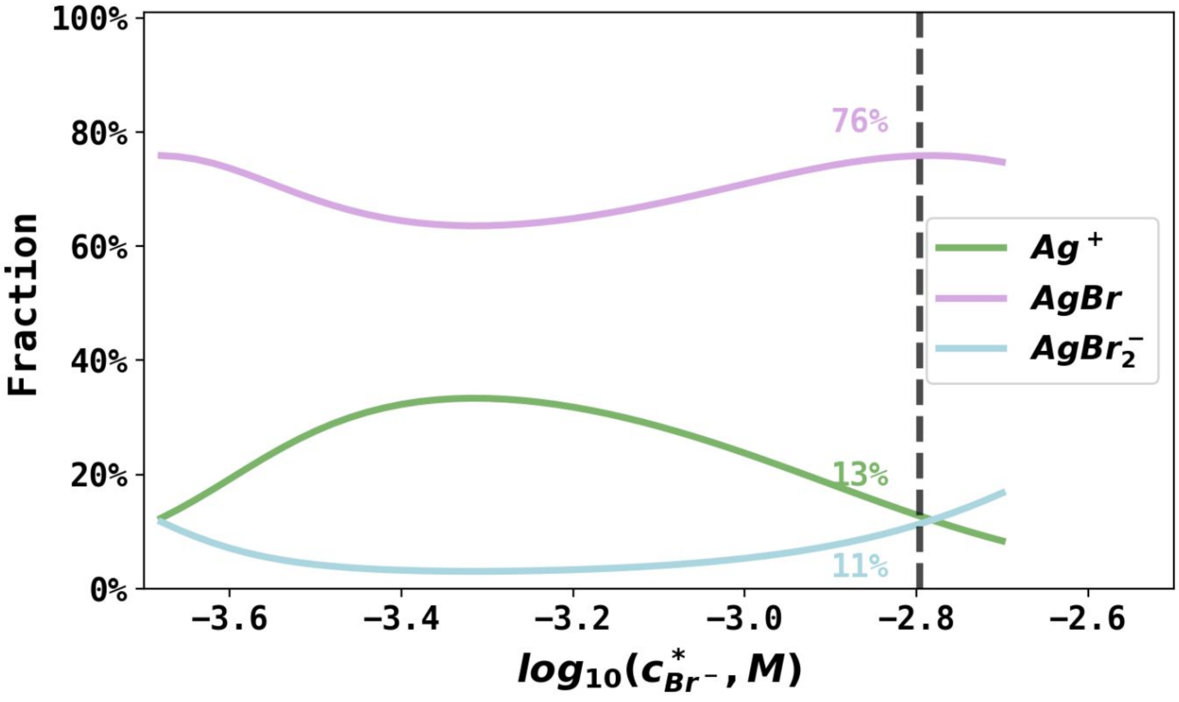

Figure S 4. Silver(I) speciation in aqueous solution showing Ag^+, $AgBr$ and $AgBr_2^-$ as a function of $\log_{10} c^*_{Br^-}$, the logarithm of bulk concentration of bromide ion using Hydra/Medusa.[5] The black dashed line and the text near it indicates the fraction of ions when total concentration of bromide is 1.6 mM. Note that the speciation shown is that of the solution phase; solid AgBr is not considered

4 AgBr stripping efficiency

By integrating the oxidative and reductive charges the fraction of stripped AgBr was obtained as a function of $c^*_{Br^-}$ and scan rates are shown in Table S 2. The fraction stripped decreases with a decrease in scan rates and decrease in bulk concentration of bromide. The trends are discussed in the main text.

Table S 2. The fraction of stripped AgBr by comparing the cathodic relative to anodic charges as a function of, $c^*_{Br^-}$, the bulk concentration of Br^- and scan rates.

	20 mV/s	50 mV/s	100 mV/s	200 mV/s	400 mV/s
**0.2 mM**	53%	51%	50%	45%	50%
**0.4 mM**	56%	56%	58%	58%	59%
**0.8 mM**	77%	75%	80%	79%	78%
**1.2 mM**	77%	80%	82%	83%	85%
**1.6 mM**	87%	89%	89%	90%	92%

5 AgBr deposit thickness

To estimate the AgBr deposit thickness during the experimental voltammetric scans, the current was integrated versus time to obtain the charge transferred, which was then used to estimate the AgBr thickness on the electrode using the following equation:

$$Thickness \approx \frac{charge}{Faraday\ Constant} \times molar\ mass \div density \div area\ of\ electrode$$

The parameters used to calculate AgBr thickness are shown in Table S 3. Figure S 5 gives an example of the estimated AgBr deposit thickness as the voltammetric scan proceeds. The summary of the peak AgBr thickness at different bromide bulk concentrations and scan rates are shown in Table S 4.

Table S 3. Parameters used to estimate AgBr thickness during the voltammetric scan.

Parameter	Values
Faraday Constant	96485 C/mol
AgBr molar mass	187.77 g/mol
AgBr density	$6.473 \times 10^6 \frac{g}{m^3}$
Area of electrode	$\pi \times (1.13 \times 10^{-3})^2\ m^2 = 4.01 \times 10^{-6}\ m^2$
Ag density	10.49 g/cm^3
Ag atomic radius	172 pm

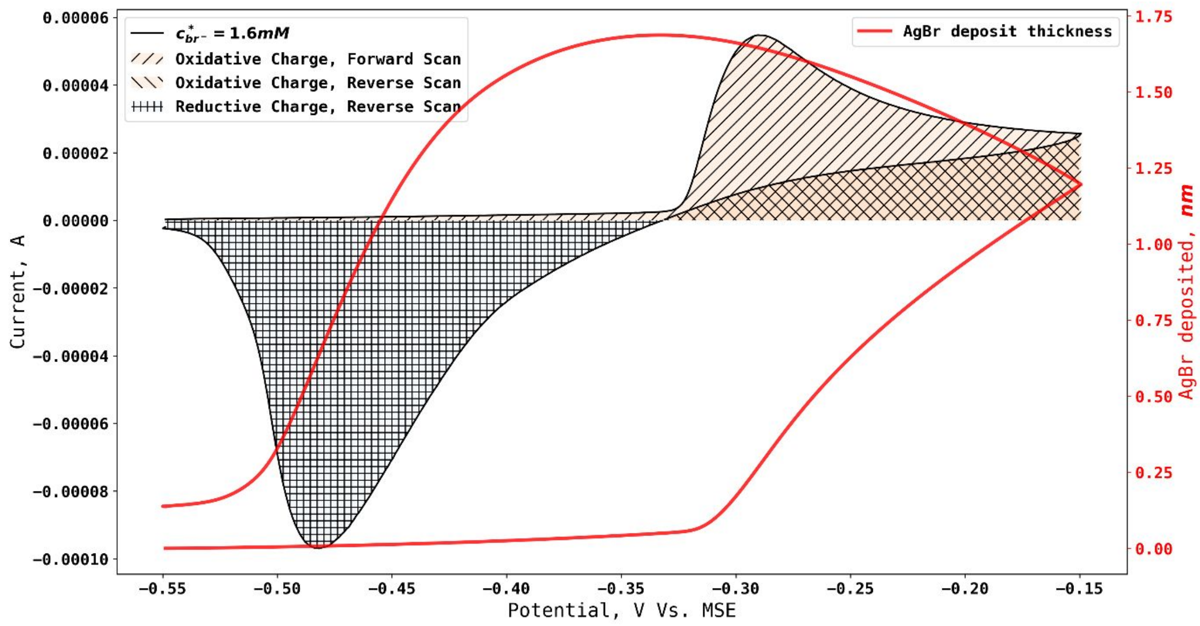

Figure S 5. The voltammetric scan when $c^*_{Br^-} = 1.6\ mM$ and $\nu = 400\ mV/s$. The black trace represents the voltammogram, and *the red* trace *represents the AgBr deposit thickness as voltammetric scan proceeds. A maximum of 1.69 nm AgBr is estimated to deposited on electrode surface during scan.*

Table S 4. The maximum AgBr deposit thickness during the voltammetry scan as a function of bulk concentration of bromide, $c^*_{Br^-}$ and scan rate, ν.

AgBr deposited, nm		scan rate, mV/s				
		20	50	100	200	400
$c^*_{Br^-}$, mM	0.2	1.69	1.16	0.79	0.55	0.36
	0.4	4.10	2.24	1.39	0.91	0.61
	0.8	5.02	3.18	2.12	1.46	1.01
	1.2	8.47	4.7	3.08	2.07	1.36
	1.6	**9.31**	**7.79**	**3.90**	**2.54**	**1.69**

6 References

1. Henstridge, M. C.; Compton, R. G., Direct extraction of kinetic parameters from experimental cyclic voltammetry. *J. Electroanal. Chem.* **2012,** *681*, 109-112.

2. Wang, Y.; Limon-Petersen, J. G.; Compton, R. G., Measurement of the diffusion coefficients of $[Ru(NH_3)_6]^{3+}$ and $[Ru(NH_3)_6]^{2+}$ in aqueous solution using microelectrode double potential step chronoamperometry. *J. Electroanal. Chem.* **2011,** *652* (1), 13-17.

3. Gavaghan, D., An exponentially expanding mesh ideally suited to the fast and efficient simulation of diffusion processes at microdisc electrodes. 1. Derivation of the mesh. *J. Electroanal. Chem.* **1998,** *456* (1-2), 1-12.

4. Compton, R. G.; Laborda, E.; Kaetelhoen, E.; Ward, K. R., *Understanding voltammetry: simulation of electrode processes*. 2nd ed.; World Scientific London, 2020.
5. Puigdomenech, I., Hydra/Medusa chemical equilibrium database and plotting software. *KTH Royal Institute of Technology* **2004**.
6. Gammons, C. H.; Yu, Y., The stability of aqueous silver bromide and iodide complexes at 25–300°C: Experiments, theory and geologic applications. *Chem. Geol.* **1997,** *137* (3), 155-173.
